# Deep multiple instance learning for predicting chemotherapy response in non-small cell lung cancer using pretreatment CT images

**DOI:** 10.1038/s41598-022-24278-3

**Published:** 2022-11-18

**Authors:** Runsheng Chang, Shouliang Qi, Yanan Wu, Qiyuan Song, Yong Yue, Xiaoye Zhang, Yubao Guan, Wei Qian

**Affiliations:** 1 College of Medicine and Biological Information Engineering, Northeastern University, Shenyang, China; 2 Key Laboratory of Intelligent Computing in Medical Image, Ministry of Education, Northeastern University, Shenyang, China; 3 Department of Radiology, Shengjing Hospital of China Medical University, Shenyang, China; 4 Department of Oncology, Shengjing Hospital of China Medical University, Shenyang, China; 5 Department of Radiology, The Fifth Affiliated Hospital of Guangzhou Medical University, Guangzhou, China

**Keywords:** Biomedical engineering, Outcomes research, Cancer imaging, Lung cancer

## Abstract

The individual prognosis of chemotherapy is quite different in non-small cell lung cancer (NSCLC). There is an urgent need to precisely predict and assess the treatment response. To develop a deep multiple-instance learning (DMIL) based model for predicting chemotherapy response in NSCLC in pretreatment CT images. Two datasets of NSCLC patients treated with chemotherapy as the first-line treatment were collected from two hospitals. Dataset 1 (163 response and 138 nonresponse) was used to train, validate, and test the DMIL model and dataset 2 (22 response and 20 nonresponse) was used as the external validation cohort. Five backbone networks in the feature extraction module and three pooling methods were compared. The DMIL with a pre-trained VGG16 backbone and an attention mechanism pooling performed the best, with an accuracy of 0.883 and area under the curve (AUC) of 0.982 on Dataset 1. While using max pooling and convolutional pooling, the AUC was 0.958 and 0.931, respectively. In Dataset 2, the best DMIL model produced an accuracy of 0.833 and AUC of 0.940. Deep learning models based on the MIL can predict chemotherapy response in NSCLC using pretreatment CT images and the pre-trained VGG16 with attention mechanism pooling yielded better predictions.

## Introduction

Globally, lung cancer is the most common cancer and is responsible for the highest incidence and death. Non-small cell lung cancer (NSCLC) is the most common subtype and accounts for 80% of all lung cancers^1^. Although the lung cancer death rate has fallen by 54% for men (after 1990) and by 30% for women (after 2002) because of improved healthcare and increased access to early screening, the 2-year and 5-year relative survival rates are still only 36% and 19%, respectively^2,3^. Furthermore, most of the diagnosed cases are at advanced stages. The individual prognosis for existing clinical treatment options such as surgery, chemotherapy, radiotherapy, targeted drug therapy, and immunotherapy is quite different, and there is an urgent need to precisely predict and assess treatment response^4^.

During clinical treatment, there are significant individual differences in patients with NSCLC. Chemotherapy is the most widely used first-line treatment for lung cancer, and the prediction of its prognostic endpoints will significantly improve clinical applications. No treatment option can completely replace chemotherapy. However, targeted drugs and immune checkpoint inhibitors (as supplements) are gaining increasing importance in treating NSCLC^5^. In clinical practice, oncologists usually judge whether to apply chemotherapy to a patient based on related guides, combined with clinical indicators, biopsy, gene sequencing, and pathological section analysis. The biopsy, gene sequencing, and pathological sectioning procedures are usually invasive. Here, only the local information of the tumor is considered, and the progress of the disease cannot be tracked^6,7^.

Radiomics has been widely used to analyze and evaluate medical images^8–10^. By extracting quantitative features from medical images, radiomics has achieved impressive performance in predicting pathological, radiological, and chemotherapeutic responses in NSCLC^11^, breast cancer^12^, and prostate cancer^13^. However, radiomics has not yet been translated into clinical practice because it usually requires a selection of the region of interest and manual extraction of features and feature selection^14^.

In recent years, deep learning models have shown great potential in providing the end-to-end solution from medical images to clinical endpoints^15–18^. These achievements benefited from the developing of deep convolutional neural networks (CNNs). The nonlinear and even high-dimensional information in medical images can be learned automatically by deep CNNs^19–22^. Xu et al. used recurrent neural network (RNN) to predict survival and cancer-specific outcomes in 179 NSCLC patients with time-series Computed tomography (CT) scans^23^. Paul et al. used a transfer learning model to extract deep features to predict short-and long-term survivors with lung adenocarcinoma and achieved an accuracy of 90%^24^.

Multiple instance learning (MIL) is a weakly supervised method and has been widely used in deep learning as it allows for labelling a series of images (bag) instead of each slice (instance)^25^. This strategy is conducive to extracting global features and can avoid the influence of individual false positive instances^26,27^. Chen et al. developed a MIL network to extract features in retinopathy images of prematurity to improve staging results^28^. Li et al. proposed a multi-resolution MIL model to detect suspicious regions for fine-grained grade prediction in 830 patients^29^. Li et al. assessed the severity level of COVID-19 on the CT images of 229 cases using a MIL framework^30^. No MIL studies predicting chemotherapy response in NSCLC have been reported.

In this study, we proposed a deep multiple instance learning (DMIL) model to predict the chemotherapeutic response of NSCLC patients using pretreatment CT images. The contributions contain three primary aspects. Firstly, the proposed DMIL model demonstrates great performance and generalizability for predicting treatment response. Secondly, different pre-trained backbone CNNs are evaluated for feature extraction. Thirdly, an attention mechanism pooling method is proposed and compared with other methods. The developed model is likely to be a weakly supervised, non-invasive, low-cost tool for NSCLC management.

## Materials and methods

### Patient cohorts

We enrolled 661 patients from the Shengjing Hospital of China Medical University. The inclusion criteria were that the patient was diagnosed with NSCLC between 2015 and 2019 and treated using chemotherapy alone as the first-line regimen. By excluding patients with no diagnosis reports or no CT images before or after chemotherapy, only 301 patients were included to generate Dataset 1. Using the same inclusion criteria, 42 patients from the Fifth Affiliated Hospital of Guangzhou Medical University were enrolled to generate Dataset 2. This study was approved by the ethics committee of the Shengjing Hospital of China Medical University and the Fifth Affiliated Hospital of Guangzhou Medical University. All experiments were performed in accordance with relevant guidelines and regulations. Waiver for informed consent was approved the ethics committee of the Shengjing Hospital of China Medical University and the Fifth Affiliated Hospital of Guangzhou Medical University because it was a retrospective study. The characteristics of patients are listed in Table 1, and the parameters for clinical CT acquisition are listed in Table 2. Dataset 1 was stratified into training (n = 211), validation (n = 30) and test (n = 60) cohorts randomly in a ratio of 70:10:20%. Dataset 2 was used as an external validation cohort to assess the generalizability of the trained models.

**Table 1:** Clinical characteristics of NSCLC patients.

Characteristics	Dataset 1			Dataset 2		
	Response group	Nonresponse group	*p* value	Response group	Nonresponse group	*p* value
Number of patients	163	138	–	22	20	–
**Gender**						
Male	89	77	3.241^a^	18	16	5.306^a^
Female	74	61		4	4	
Age [years] (Mean ± S. D.)	63.59 ± 11.28	65.02 ± 10.63	0.662^b^	65.86 ± 9.19	63.48 ± 13.34	0.841^b^
**Histological type**						
Adenocarcinoma	132	108	1.378^a^	12	13	2.251^a^
Squamous cell carcinoma	31	30		10	7	
**Smoking status**						
Ever	61	79	2.306^a^	16	15	1.436^a^
Never	102	59		6	5	
Number of treatment courses (Mean ± S. D.)	4.86 ± 1.25	3.64 ± 2.82	1.255^b^	3.74 ± 1.67	3.16 ± 1.89	0.841^a^

^a^*p* value for the Chi-square test; ^b^p-value for the two-sample *t *test.

**Table 2:** Parameters for CT image acquisition.

Parameter	Dataset 1	Dataset 2
kVp [kV]	120	120
X-ray tube current (mean ± S.D.) [mA]	226.24 ± 66.86	254.25 ± 54.48
Slice thickness [mm]	2.5 (*n* = 19); 3.0 (*n* = 262); 5.0 (*n* = 20)	2.0 (n = 42)
Pixel size [mm]	0.74 ± 0.15	0.85 ± 0.67
CT scanner manufacturer	GE Medical (*n* = 12), Siemens (*n* = 12),	
Toshiba (*n* = 15), Philips (*n* = 262)	Siemens (*n* = 42)	

The gold standard for measuring the axial diameter of lesions and assessing clinical prognosis is called the response evaluation criteria in solid tumors (RECIST)^31^. By comparing CT images before and after chemotherapy, all cases were categorized as “response” or “nonresponse.” The response group includes CR (complete response) and PR (partial response), whereas the nonresponse group includes PD (progressive disease) and SD (stable disease). According to RECIST, all tumors were independently assessed by two experienced radiologists, and they reached an agreement through discussion if there was a disagreement. For Dataset 1, the interval between CT scans before and after chemotherapy was 4.74 ± 1.36 (response) and 3.96 ± 2.55 (nonresponse) treatment courses (3-week treatment course), respectively. Whereas, the interval was 3.21 ± 1.65 (response) and 3.33 ± 1.24 (nonresponse) treatment courses for Dataset 2.

### Model design

**Figure 1:**
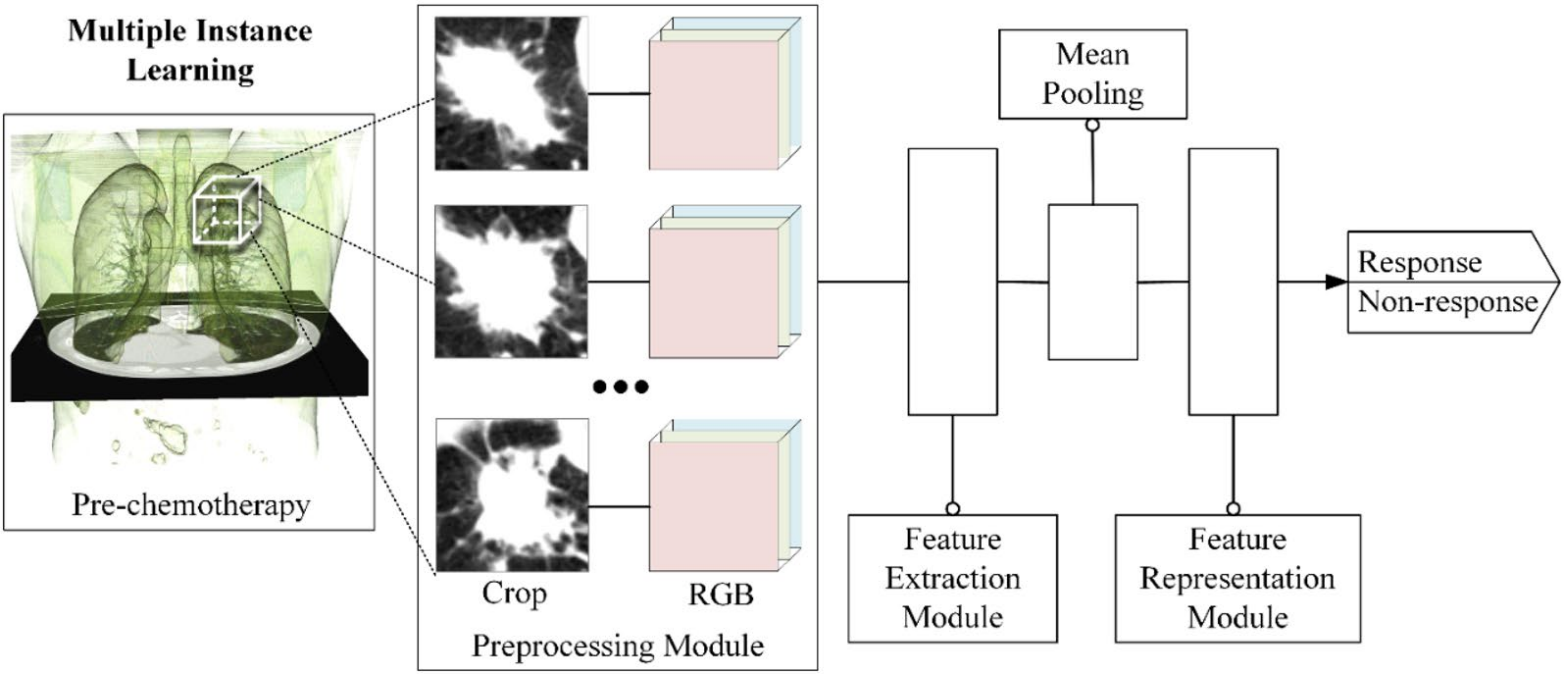
Flowchart of the experiment.

An illustration of the proposed DMIL model is provided in Fig. 1. It consists of three main modules:

1. Preprocessing module
  The CT images of NSCLC patients have different slice thicknesses and pixel sizes because of the different acquisition equipment. To eliminate the influence of such variables on the model prediction, we resampled the CT voxels to an isotropic resolution of 1 x 1 x 1 mm^3^. Considering the accuracy and efficiency of subsequent CNN operations, a cuboid of 64 × 64 × 32 was cropped from CT images to contain the whole tumor. Two experienced radiologists performed this operation after reaching an agreement. A tumor cuboid is treated as a bag, and each patch (or slice) within the cuboid is denoted as an instance. Next, using the grayscale CT images as input to the pre-trained CNN, we converted the images to RGB format and resized each layer to adapt to different pre-trained networks. Each cuboid or bag was marked “response” or “nonresponse.”
2. Feature extraction module
  Each instance is input into a path of the model, in which a deep CNN backbone network is used to extract the features. The backbone network is pre-trained by images from ImageNet^32^ and fine-tuned on our CT slices. Five baseline pre-trained models (AlexNet, VGG16, ResNet34, DenseNet, and MobileNetV2)^33–37^ are evaluated. The weights in backbone networks are shared across different paths. A mean pooling method was applied to reduce the dimension of extracted features to 32 × 512.
3. Feature representation module
  Three methods (max pooling^38^, convolutional pooling, and attention mechanism pooling^39^) were used to represent the features of a bag into a fully connected layer and then classify it into response and nonresponse categories. Through the combination of five pre-trained backbone networks with transfer learning and three pooling methods, several models were obtained and compared.

### Neural network structure

Pre-trained models like VGG16 are widely used to extract features from images because of their simple structure and good generalization performance in transfer learning. All weights were trained in the set of 14 million 2D color images called ImageNet^32^. The network structure was implemented in Python 3.9, using PyTorch 1.10^40^.

**Figure 2:**
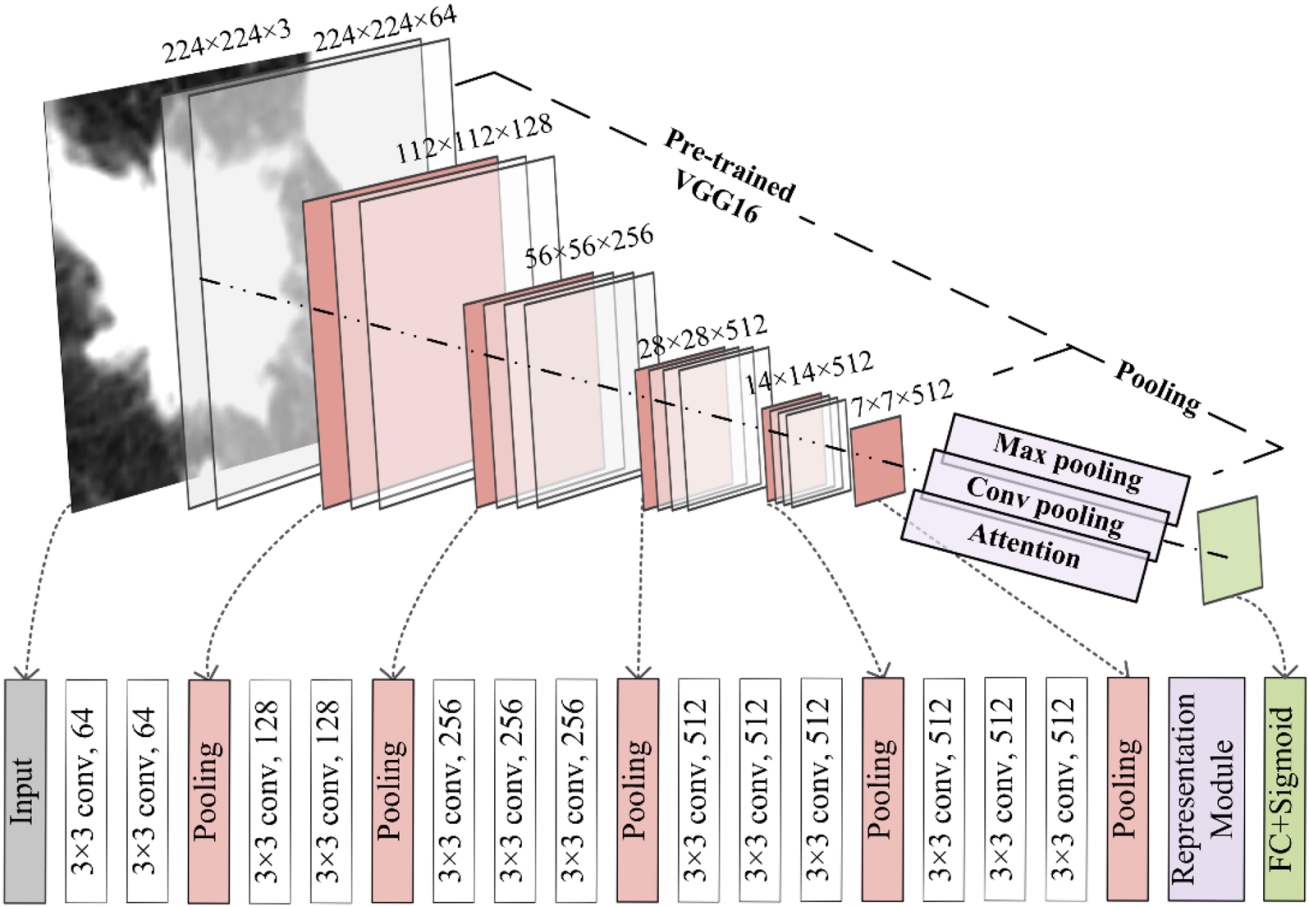
Structure of the pre-trained VGG16 for feature extraction.

Figure 2 shows that VGG16 has 5 convolutional layers, each segment has 2 or 3 convolutional layers, and a maximum pooling layer is connected at the end of each segment to reduce the image size. The number of convolutional kernels in each segment is the same, and the closer to the fully connected layer, the more convolutional kernels there are. The last segment is a fully connected network, and we added a pooling segment to represent features before the fully connected segment.

The specific process of network structure is given as follows. Firstly, each CT slice from one patient (32 × 224 × 224) was passed (in order) through the feature extractor based on VGG16. A 32 × 512 × 7 × 7 tensor was then obtained, and it contained features for each slice. Secondly, a global average pooling layer was applied to reduce the tensor dimension to 32 × 512. Thirdly, we input this tensor into the feature representation module.

**Figure 3:**
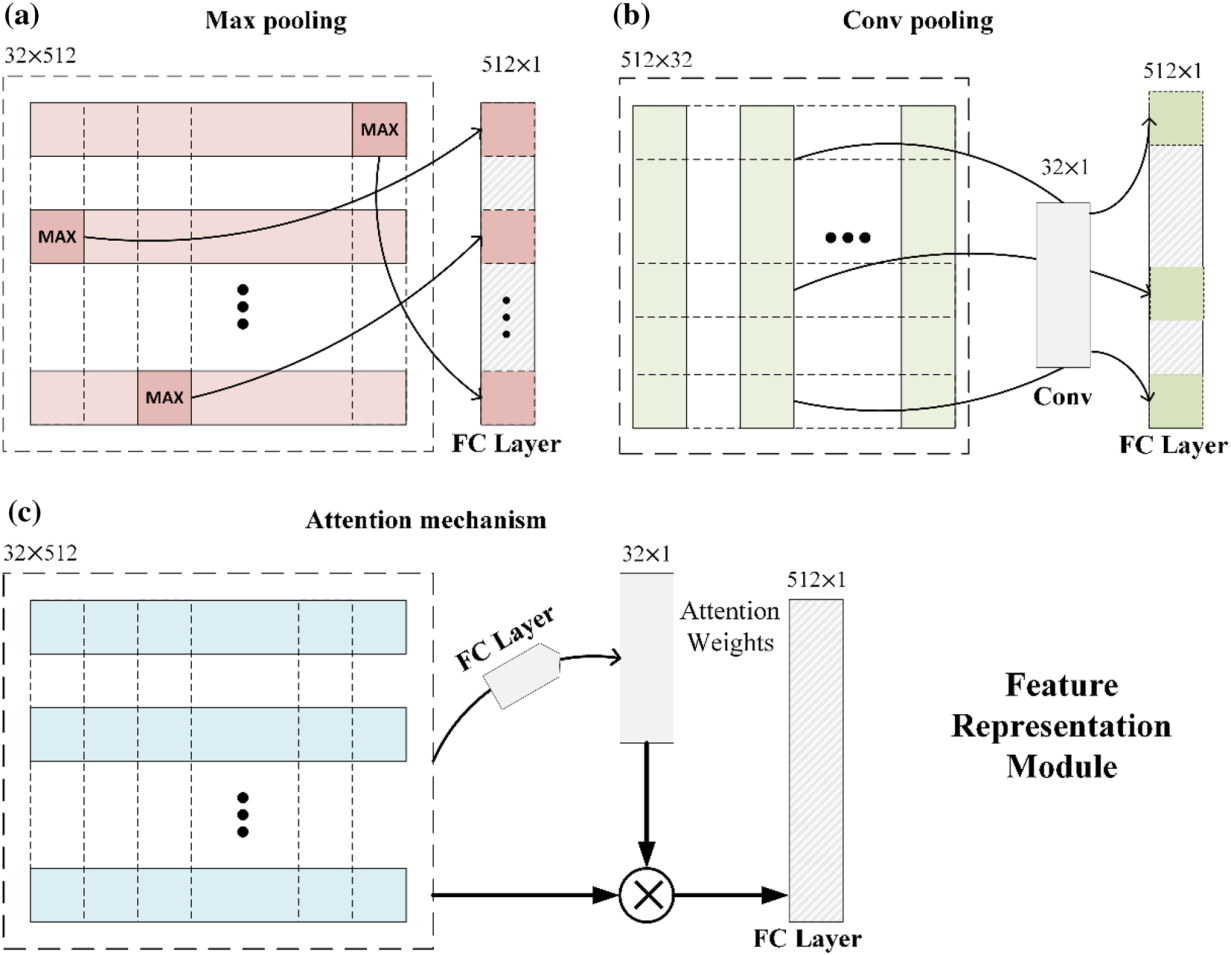
Feature representation module. (**a**) Structure of the max pooling method; (**b**) Structure of the convolutional pooling method; (**c**) Structure of the attention mechanism pooling method.

Three methods were used to represent the features of a bag:

1. Max pooling
  A max pooling was applied across slices to obtain a 512-dimensional vector, and this operation was performed between slices. Among the 512 kernels containing features, the largest feature in every 32 slices is taken to represent one feature kernel. Then it is passed to a fully connected layer, and the sigmoid activation function is used to obtain a prediction in the 0 to 1 range (Fig. 3a).
2. Conv pooling
  A 32 × 1 convolutional layer was applied to this tensor and obtained a 512-dimensional vector. This convolutional layer is applied to obtain a feature vector that can characterize each convolution kernel using a convolution operation (Fig. 3b).
3. Attention mechanism pooling
  This DMIL design is commonly referred to as "Attention-based multiple instance learning", as presented in the study by Ilse et al.^41^. Two fully connected layers were applied to obtain a 32 × 1 vector called attention weight and then it was multiplied by the 32 × 512 tensor to obtain a 512-dimensional vector (Fig. 3c). This attention mechanism pooling determines the weights to instances by a neural network. Additionally, the sum of all weights must be equal to 1 for a bag-level prediction. The weighted average meets the requirements that the weights and the embedding are part of the *f* function. Let $\mathrm{H}=\left\{{\mathrm{h}}_{1},\dots ,{h}_{k}\right\}$ be a bag of $\mathrm{k}$ embeddings, and then we propose the following MIL pooling:
  1 $$\mathrm{z}=\sum_{k=1}^{k}{a}_{k}{h}_{k}$$
  where
  2 $${a}_{k}=\frac{exp\left\{{w}^{T}\mathrm{tanh}(V{h}_{k}^{T})\right\}}{{\sum }_{j=1}^{k}exp\left\{{w}^{T}\mathrm{tanh}(V{h}_{j}^{T})\right\}}$$

### Statistical analysis

Performance measures for each model included the accuracy, sensitivity, specificity, F1-score, the area under the receiver operating characteristic curve (AUC), and confusion matrix. The decision curve analysis (DCA) was also used to evaluate each model by quantifying the benefit at different threshold probabilities. We also provided a 95% confidence interval (CI) for AUC, the cut-off using Youden’s index, and the shortest distance from the coordinate (0, 1) for the ROC curve to assess the variability in estimates.

To assess whether these findings were dependent on the clinical features, we performed a two-sample t-test to compare the age and number of treatment courses and a chi-square test to compare the gender, histological type, and smoking status between the response and nonresponse groups. The significance level was set to 5%.

## Results

### Clinical characteristics

The information on clinical characteristics is shown in Table 1. In Dataset 1, there were no significant differences between the response and nonresponse groups for the measures of gender, age, histological type, smoking status, and the number of treatment courses. Similarly, no significant differences were observed between the two groups in Dataset 2.

### Performance of the DMIL model

**Figure 4:**
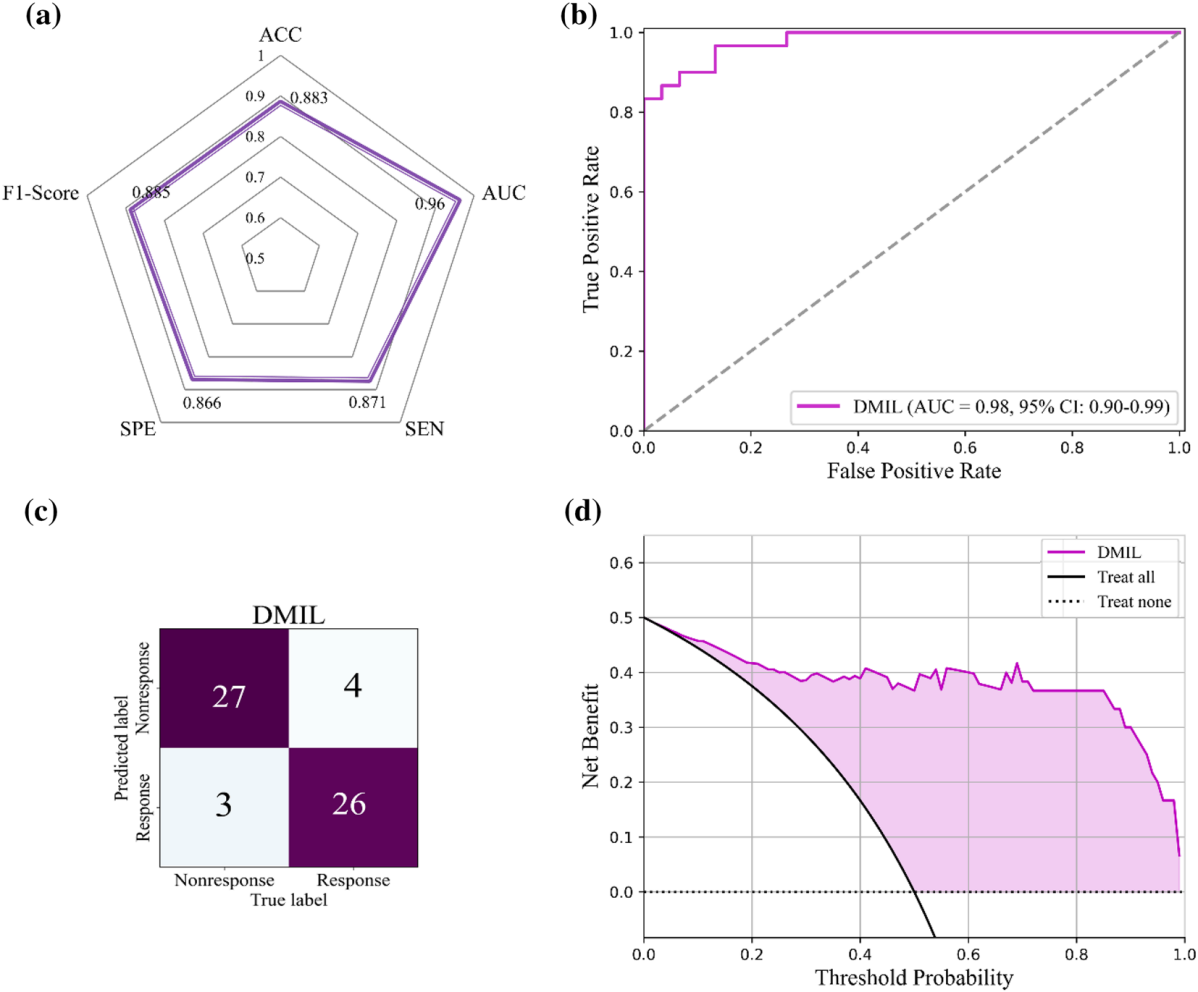
Performance of the DMIL model in the test cohort. (**a**) Statistical results; (**b**) The ROC curve; (**c**) The confusion matrix; (**d**) The DCA result.

The performance of the DMIL model is shown in Fig. 4 and Table 3. As the radar chart demonstrates in Fig. 4a, the accuracy, AUC, sensitivity, specificity, and F1-score were 0.883, 0.982, 0.871, 0.867, and 0.885, respectively, whereas the cut-off value was 0.833. Figure 4b,c show the ROC and confusion matrix. The model correctly predicted 27 of 30 nonresponse and 26 of 30 response patients in the test cohort. According to the DCA shown in Fig. 4d, the DMIL curve showed much more net benefit than the baseline curves of “Treat none” and “Treat all”.

**Table 3:** Performance of the DMIL models with different backbone networks.

Backbone	Pooling	ACC	AUC	SEN	SPE	F1-Score
AlexNet	Max	0.848	0.937	0.862	0.867	0.847
	Conv	0.817	0.922	0.828	0.833	0.814
	AM	0.866	0.951	0.892	0.900	0.862
VGG16	Max	0.850	0.958	0.800	0.767	0.862
	Conv	0.817	0.931	0.788	0.767	0.825
	AM	0.883	0.982	0.871	0.867	0.885
ResNet34	Max	0.783	0.856	0.793	0.800	0.779
	Conv	0.800	0.886	0.781	0.767	0.806
	AM	0.816	0.864	0.771	0.733	0.831
DenseNet	Max	0.817	0.883	0.828	0.833	0.814
	Conv	0.783	0.892	0.775	0.767	0.787
	AM	0.850	0.927	0.889	0.900	0.842
MobileNetV2	Max	0.800	0.871	0.765	0.733	0.813
	Conv	0.817	0.907	0.771	0.733	0.831
	AM	0.850	0.963	0.784	0.733	0.866

*AM attention mechanism, *ACC* accuracy, *AUC* area under the curve, *SEN* sensitivity, *SPE* specificity.

### Comparison with the counterparts

**Figure 5:**
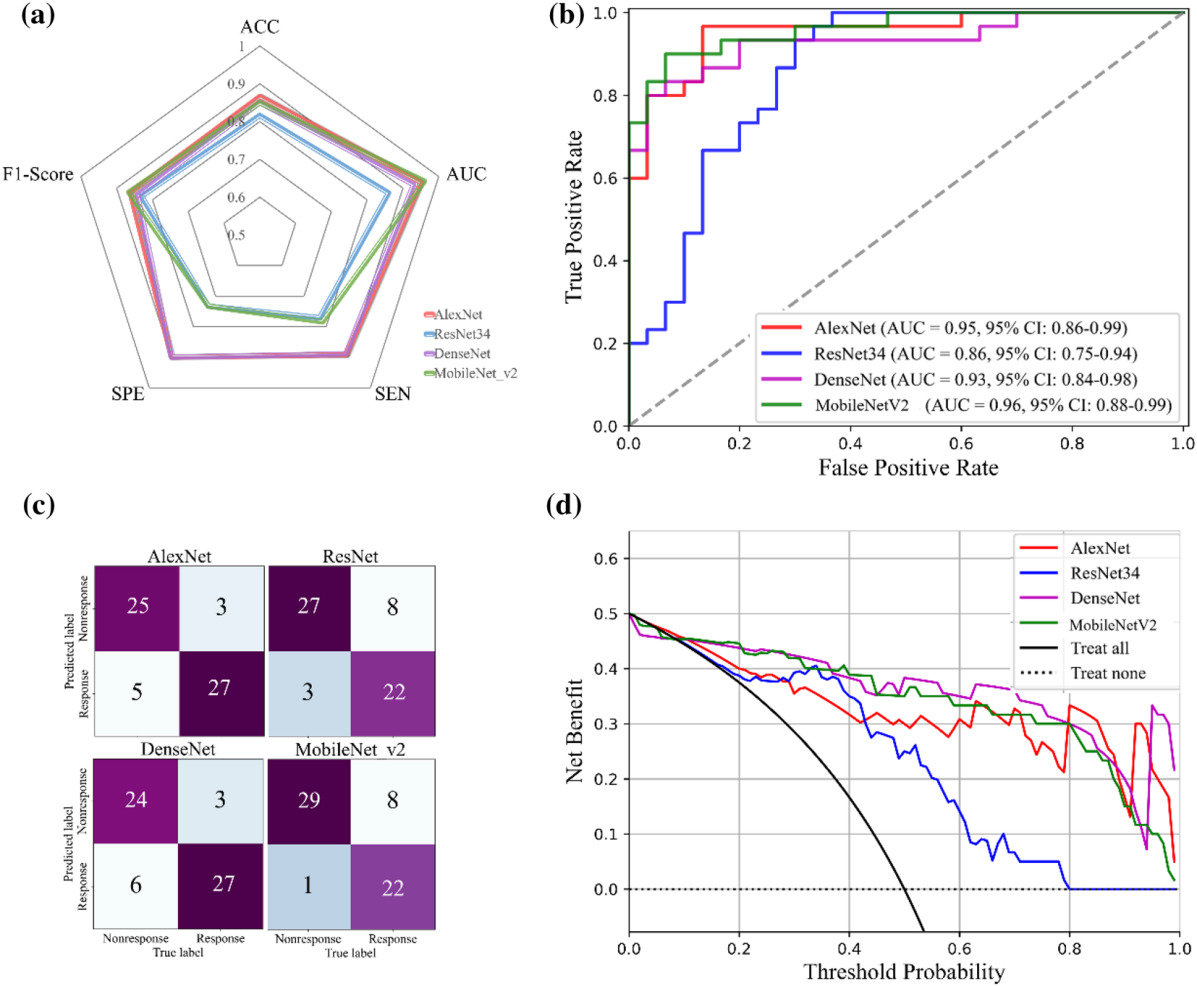
Performance of four counterparts with different backbone networks for feature extraction. (**a**) Statistical results; (**b**) The ROC curve; (**c**) The confusion matrix; (**d**) The DCA result.

Five different pre-trained models were investigated in the feature extraction module while using the attention mechanism pooling method. The performance of the four counterparts is shown in Fig. 5 and Table 3. The accuracy, AUC, sensitivity, specificity, and F1-score are shown in the radar chart (Fig. 5a). The ROC and confusion matrix are shown in Fig. 5b,c, the AUC of AlexNet, ResNet34, DenseNet and MobileNet_v2 were 0.951, 0.864, 0.927 and 0.963, respectively. The DMIL model had a higher AUC than the four backbone models. This finding was confirmed by DCA (Fig. 5d) and proves that the DMIL model is more robust than the four backbone models.

**Figure 6:**
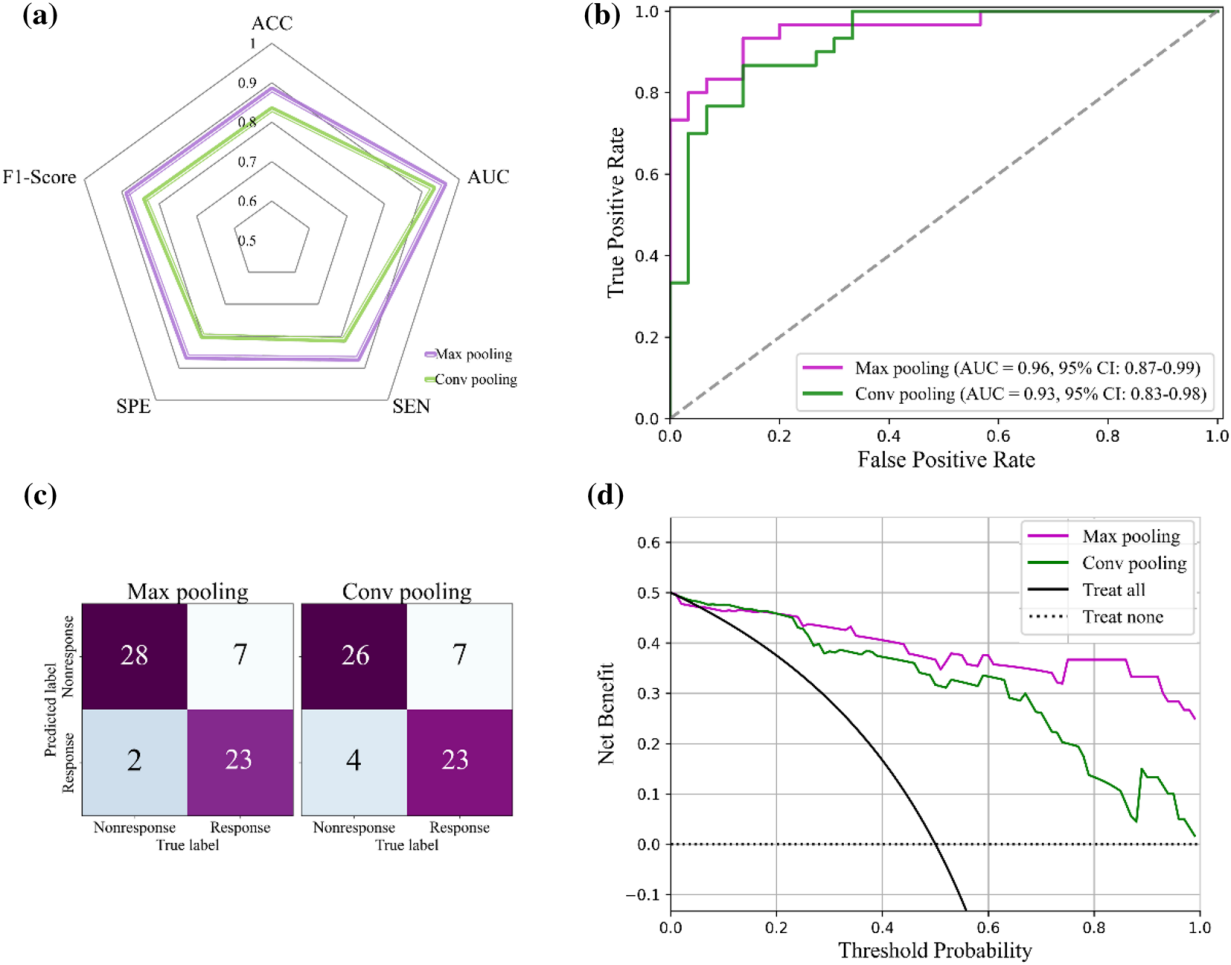
Performance of the two counterparts with different representation modules. (**a**) Statistical results; (**b**) The ROC curve; (**c**) The confusion matrix; (**d**) The DCA result.

Three pooling methods combined with the VGG16 model were studied in the feature representation module. The performance of the two counterparts is shown in Fig. 6 and Table 3. The accuracy, AUC, sensitivity, specificity, and F1-score are shown in the radar chart of Fig. 6a. The ROC and confusion matrix are shown in Fig. 6b,c, and the AUC of max pooling and convolutional pooling methods were 0.958 and 0.931, respectively. Compared with the DMIL model, both pooling methods had a lower AUC. In the DCA (Fig. 6d), the DMIL model had better performance than both pooling methods.

During the model training, the Adam optimizer was utilized. The learning rate, batch size, and the number of epochs were set as 10–5, 32, and 50, respectively.

### Performance in the external validation dataset

**Figure 7:**
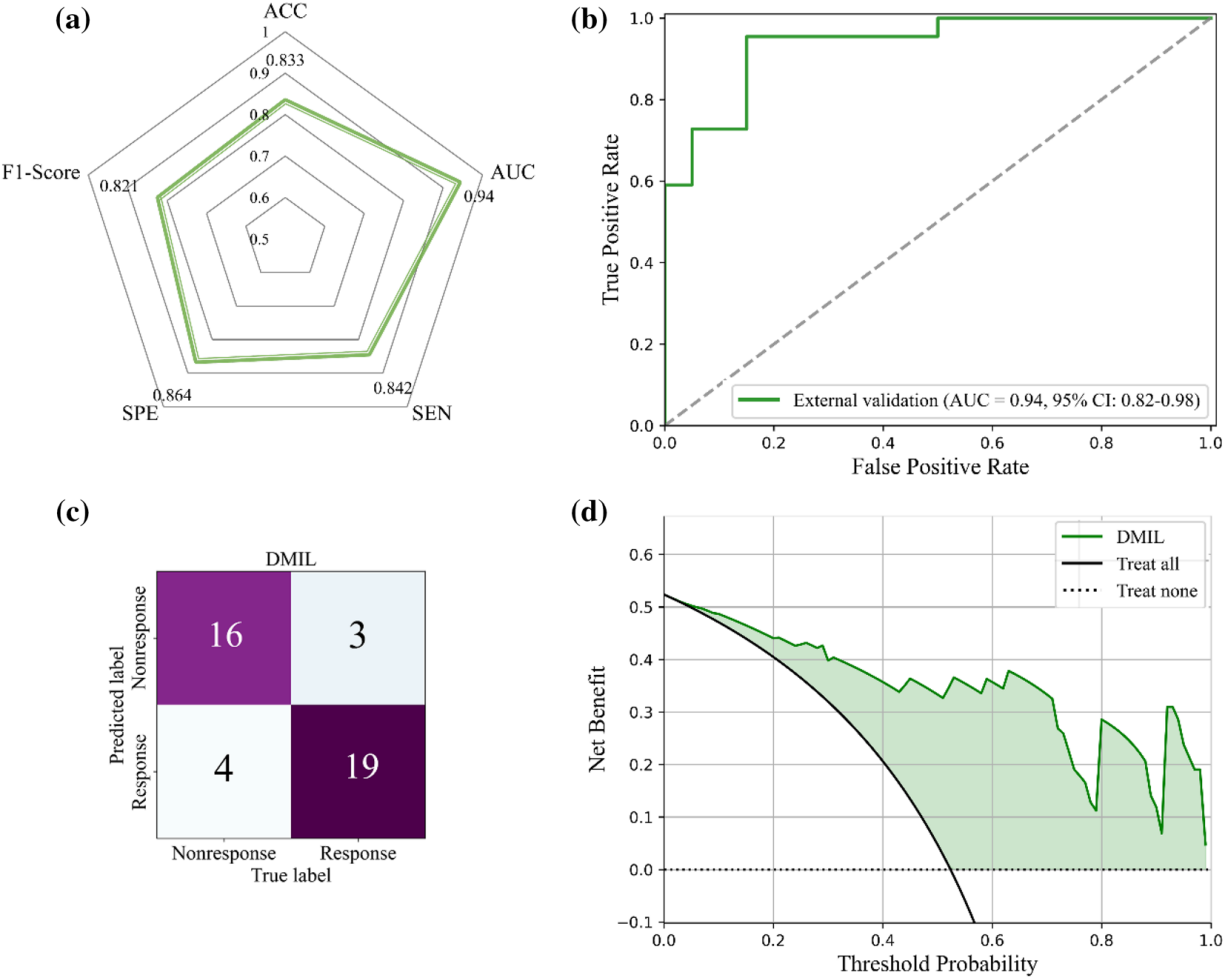
Performance of the DMIL model in the external validation. (**a**) Statistical results; (**b**) The ROC curve; (**c**) The confusion matrix; (**d**) The DCA result.

In the external validation cohort (Dataset 2), 42 NSCLC patients were used to validate the DMIL model, and the performance is shown in Fig. 7. As the radar chart shows in Fig. 7a, the accuracy, AUC, sensitivity, specificity, and F1-score were 0.833, 0.940, 0.842, 0.864, and 0.821, respectively, using a cut-off value of 0.805. The ROC and CM are shown in Fig. 7b,c, and the model correctly predicted 17 of 20 nonresponse and 18 of 22 response patients. The DCA results in Fig. 7d indicated that the curve of the DMIL model was higher compared with the treat none and treat all groups.

## Discussion

In this study, a deep learning model based on MIL was proposed to predict chemotherapy response in NSCLC patients using CT images. The model with pretrained VGG16 backbone and an attention mechanism pooling method demonstrated the best performance, comparing 15 different designs. Furthermore, good generalizability was observed when using an external validation dataset.

A weakly supervised method called multiple instance learning was used to train the model. Each patient in the dataset is regarded as a bag, and each bag contains all slices of one patient, which is regarded as an instance. Each bag has a training label, and the instances in the bag are unlabeled. A bag is given a positive label if at least one positively-labeled instance is in the bag. For a negatively-labeled bag, all its instances are negatively-labeled^41,42^. The advantage of this weakly supervised learning method over supervised learning is that not every CT slice of the patient is adequate for evaluating the efficacy of chemotherapy^27^. Training labels based on the bag instead of instances can eliminate the noise that has no predictive effect on the chemotherapy effect and reduces the false positives in the training process^43^.

Using pre-trained models as the backbone module for computer vision and natural language processing tasks is popular in deep learning. Because the development of neural network models for these tasks requires a lot of computational resources and time, pre-trained models can considerably boost related works. There are three main advantages to pre-trained models: (a) better initialization: the initial skill in the source model is higher; (b) faster convergence: the rate of improvement in skill is steeper during training of the source model than in other cases; (c) higher asymptote: the fusion skill of the trained models is better than the other cases^44–47^. We used five different pre-trained models (AlexNet, VGG16, ResNet34, DenseNet, and MobileNet_v2) as the backbone module to extract features from CT slices. We found that VGG16 had better predictive performance than the other models. This model is characterized by stacking several convolutional layers and pooling layers, and the convolutional layers and pooling layers both use the same convolution kernel and pooling kernel parameters, which can easily form a deeper network structure. Overall, VGG16 has small filters and deeper networks.

We tried three strategies to represent features in the pooling module: max pooling, convolutional pooling, and attention mechanism pooling. For the max pooling method, the features of one bag are represented by taking the maximum value of all layers in each convolutional kernel^48^. This may lose important information that is not the maximum value. For the convolutional pooling method, the information of all slices in one bag is calculated to represent global features. However, this single-layer convolution is not fed back to the network before, so the critical information may also be lost^49^. The attention mechanism pooling method combines a fully connected and attention mechanism. Using two fully connected layers, this method can obtain the weights of all layers for each convolution kernel^50^. After this, multiplying the weights with the previous features produces a matrix representing the global features. This feedback mechanism can update and optimize the network parameters while preserving the features of each slice^51,52^.

After further validation, this DMIL model might be applied to clinical practice. Firstly, clinical oncologists can make personalized treatment plans for patients according to the predicted possibilities of response or nonresponse to the chemotherapy. If the possibility of nonresponse is very high, combined chemotherapy, radiotherapy, immunotherapy, or other programs should be considered with priority^53^. Secondly, this model is non-invasive and low-cost by utilizing the prechemotherapy CT images scanned in clinical routines. It can be extended to patients who are of low economic affordability and cannot tolerate invasive measurements^54^. Thirdly, this model is proven to have good generalizability, indicating that different hospitals and regions can introduce the model without fine-tuning.

Despite the excellent performance, there are some limitations in this research. Firstly, the five models applied in this study were all commonly used baseline architectures. More modern architectures should be tested with our proposed DMIL design in future studies. Secondly, the slices of CT images input to the model were clipped. We could try raw CT images as the input to see if it benefits the performance in future studies. Thirdly, unlike radiomic features, the features obtained by the feature representation module were unexplainable because the features extracted by VGG-16 from all instances have been fused and represented once again.

## Conclusion

Deep learning models based on MIL can predict chemotherapy response in NSCLC using pretreatment CT images. In this model, the pre-trained VGG16 through transfer learning can efficiently extract features from each instance, and an attention mechanism pooling module can estimate the importance of different instances and represent features of the bag. These excellent feature extraction and representation methods enable good model performance and generalization ability. After further clinical validation, the developed model is likely to be a weakly supervised, non-invasive, low-cost tool for NSCLC management.

## Data Availability

The datasets used in this study are available on reasonable request to Shouliang Qi (qisl@bmie.neu.edu.cn).
